# The Shared Origins of Embodiment and Development

**DOI:** 10.3389/fnsys.2021.726403

**Published:** 2021-08-17

**Authors:** Peter J. Marshall, Troy M. Houser, Staci M. Weiss

**Affiliations:** ^1^Temple University, Philadelphia, PA, United States; ^2^University of Cambridge, Cambridge, United Kingdom

**Keywords:** embodiment, evolution, development, enactivism, experience

## Abstract

As a domain of study centering on the nature of the body in the functioning of the individual organism, embodiment encompasses a diverse array of topics and questions. One useful organizing framework places embodiment as a bridge construct connecting three standpoints on the body: the form of the body, the body as actively engaged in and with the world, and the body as lived experience. Through connecting these standpoints, the construct of embodiment shows that they are not mutually exclusive: inherent in form is the capacity for engagement, and inherent in engagement is a lived perspective that confers agency and meaning. Here, we employ this framework to underscore the deep connections between embodiment and development. We begin with a discussion of the origins of multicellularity, highlighting how the evolution of bodies was the evolution of development itself. The evolution of the metazoan (animal) body is of particular interest, because most animals possess complex bodies with sensorimotor capacities for perceiving and acting that bring forth a particular sort of embodiment. However, we also emphasize that the thread of embodiment runs through all living things, which share an organizational property of self-determination that endows them with a specific kind of autonomy. This realization moves us away from a Cartesian machine metaphor and instead puts an emphasis on the lived perspective that arises from being embodied. This broad view of embodiment presents opportunities to transcend the boundaries of individual disciplines to create a novel integrative vision for the scientific study of development.

Researchers interested in development occupy niches in various disciplines including biology, psychology, neuroscience, education, and various parts of the social sciences. However, each type of expert spends much of their time viewing developmental questions through a narrow disciplinary lens. We suggest that new insights can be gained by employing the concept of *embodiment* as a wide-angle lens that can reveal novel opportunities for connecting the study of development across a diversity of domains of study. By embodiment, we refer to the nature of the body in the functioning of the individual organism. We follow Overton (2008), who proposed that “Embodiment is a concept of synthesis, a bridge that joins broad areas of inquiry into a unified whole” (p. 3). He further suggested that one way to view embodiment is as a bridge between three standpoints: the form of the body (in the sense of bodily morphology), the body as actively engaged with the world, and the body as lived experience (see also Johnson, 2007).

Here, we take a broad view, framed by the three standpoints of Overton (2008), to highlight how embodiment and development are deeply intertwined, with the hope of encouraging developmental scientists to engage with the implications of an embodied approach. We begin with the evolutionary origins of bodily form and why and how bodies arose. Given that the body does not exist separately from the actions and activities of the individual, we then move to consider the capacity for embodied engagement by the developing organism. The origins of the metazoan (animal) body are of particular interest, given that the evolution of animals involved the emergence of complex bodies with nervous systems and sensorimotor capacities that bring forth a particular sort of embodiment. However, we also emphasize that the thread of embodiment runs through all living things: From single cells, through simple multicellular entities, to the more complex bodies of animals, plants, and fungi, living things share an organizational property of self-determination that confers them with a form of autonomy which is fundamentally different from nonliving things. As such, living systems have a viewpoint or lived perspective that arises through their organization and the nature of their embodiment. This realization moves us away from a Cartesian separation of life and mind and instead puts an emphasis on the embodied perspective of the organism that arises through the nature of the organization of living things. It also underscores how embodiment as a bridge construct can lead to a synthesis of perspectives on the body, challenging us to develop new lines of interdisciplinary developmental research.

## The How and Why of Bodies

Although there cannot be a single explanation for why bodies arose, the range of opportunities that emerge from living at a larger scale likely influenced the evolution of the processes involved in the development of bodies. Simply put, the size of unicellular organisms imposes constraints that can only be overcome by becoming multicellular. Sterling and Laughlin (2017) discuss the unicellular eukaryote *Paramecium*, which ingests other, smaller microorganisms, and uses cilia for locomotion. They note that the behavioral repertoire and capacity for learning of Paramecium are limited by its size (0.05–0.30 mm), such that “the cell is still so small that locomotion must be slow, and the environment remains so evanescent that richer behavior and longer memory offer no advantage” (p. 20). From this perspective, greater size is a route towards richer and more flexible behavioral capacities, at the same time allowing access to new niches and energy flows–although this in turn also exposes the organism to new vulnerabilities.

The evolution of multicellularity is a large and complex topic that can only be touched on here. One key distinction is between simple forms of multicellularity such as filaments, clusters, balls, or sheets of cells, and the more complex bodies of animals, plants, and fungi. Simple multicellularity has evolved many times across the domain of eukaryotes, and can even be found in some prokaryotes such as cyanobacteria (Bonner, 1998). What sets more complex bodies apart is that in addition to cells adhering together, there is elaborate intercellular communication, differentiation of cells into different types and tissues, and the involvement of programmed cell death (Knoll, 2011). Behind these general commonalities are important differences in the specific evolutionary and developmental processes that shape multicellularity in animals, plants, and fungi (Niklas and Newman, 2020). For present purposes, the main point is that is that considering the evolution of complex bodies brings the concept of development to center stage. Organisms that exhibit simple multicellularity may show changes across their life cycle, as do some unicellular organisms (Driks, 2002; Huang and Hull, 2017). At some level, such changes may be construed as developmental, although they do not involve the differentiation and integration of multiple types of cell, intricate communication between cells, or the death of cells as part of the developmental process. In this sense, the evolution of complex bodies–which involves all of these characteristics–was the evolution of development itself.

It is notable that the outlines of the physiological processes involved in cell adhesion, cell–cell signaling, and cellular differentiation can be seen in the physiology of unicellular organisms, suggesting that the evolution of development did not involve the wholesale generation of new mechanisms (Grosberg and Strathmann, 2007; Sebé-Pedrós et al., 2017). Recent lines of research have extended this line of thinking to consider how the capacities of single cells can shed light on the building blocks of cognition (Levin et al., 2021; Lyon et al., 2021). We raise these points to emphasize that reaching a deeper understanding of embodiment and development involves a wide-angle outlook that extends across the breadth of living systems, even down to the level of single-celled organisms.

## Developmental and Evolutionary Pathways to Complex Bodies

One key aspect of the development of complex bodies is the differentiation of cells into different types, as parts of bodily subsystems (e.g., tissues and organs) that become integrated in support of the functioning of the whole organism. In turn, the extent of cellular differentiation in a body is tied to increasing size. Many simple multicellular eukaryotes are so small that each cell can be in contact with the external medium, but this is simply not possible in larger bodies. Beyond a certain (very small) size, wider considerations arise around metabolism and transport, which in turn require cells to take on specialized functions (Knoll, 2011; Brunet and King, 2017). In the case of animals, a range of abilities and functions arise through such specialization, including coordinated locomotion, feeding, waste excretion, and reproduction, as well as sensing of the environment–including registering the presence of other organisms.

Understanding the processes that influence the number of cell types in a body is an active area of research (Bush et al., 2017). The differentiation of cells into different types allows bodies to exist at a higher level of complexity than the individual cells of which they are comprised (Michod and Roze, 1997), with the relative complexity of a body reflected by the number of cell types that develop within it (Márquez-Zacarías et al., 2020). Within the realm of animals, some vertebrates may have as many as 250 different types of cells, while animals in basal phyla such as Cnidarians (jellyfish and corals) have far fewer. The pathway to bodily complexification in animals arose in part through the evolution of gastrulation, a series of changes in early embryonic development that gives rise to differentiated layers of cells inside the blastocyst. Although specific details are debated (Nakanishi et al., 2014; King and Rokas, 2017), the evolution of this phase of embryonic development laid the foundation for complex bodies and for the kind of embodiment that accompanies animal life.

Insights into the evolutionary origins of more complex animal bodies can be gleaned from studying basal animal phyla that have relatively simple bodies, without judgements about the nature or value of complexity (Dunn et al., 2015). Cnidarians are particularly informative in this respect: They have some degree of cellular differentiation, have multiple types of tissue within their body, and they have a nervous system, although they lack a brain. They also differ from more complex animals in having radial symmetry, rather than the bilateral symmetry that characterizes almost all other animals. In this respect, it is notable that the bodies of Cnidarians arise from two germ layers (endoderm and ectoderm) that differentiate during gastrulation, whereas more complex animal bodies arise from three germ layers. The evolution of the third germ layer (mesoderm) as a product of gastrulation was associated with a rise in bodily complexity, not only in terms of facilitating an increase in the number of cell types, but also by enabling the development and evolution of major changes in body morphology such as body cavities and bilateral symmetry (Carroll, 2001).

## The Engaged Body

Discussions of the evolution of the animal body often emphasize the important of the “Cambrian explosion” that began around 535 million years ago and left a burst of animal bodies in the fossil record. These bodies were variations on a particular body configuration involving bilateral symmetry (with an anterior and posterior end), segmentation, and specialized appendages. While the origins of this configuration predate the Cambrian, the evolution of the metazoan body shifted animals towards a new way of being in the world. The evolution of animal bodies was accompanied by an increase in the complexity and flexibility in the capacity to act, supported by the evolution of nervous systems, ultimately including brains. As multicellular animals became larger, nervous systems may have first arisen for purposes of internal coordination among cells, prior to the evolution of brains (Keijzer and Arnellos, 2017; Arendt, 2021). Networks of neurons then became incorporated into newly evolving sensorimotor systems that became connected to ways of moving and acting. This connection thus takes us from the first standpoint of bodily morphology to the standpoint of the engaged body and the notion of embodied action.

One key tenet of embodiment is that action is not the “output” of cognitive processing that is part of a stepwise perception-cognition-action sequence. Indeed, the origins of the field of embodied cognition can be traced to dissatisfaction with this mechanical view (Varela et al., 1991). Embodiment presents a distinct challenge to a view of the organism as a passive recipient of “information” with prespecified meaning that is then subjected to computational processing, followed by a behavioral response. Embodied treatments instead emphasize the active nature of the individual organism, with recent accounts focusing on how agency arises through the prospective nature of cognitive processes (Clark, 2013). This emphasis on anticipatory, future-oriented processes takes us from a reactive, feedforward, homeostatic view of organismic functioning to a view in which *allostasis* is the predominant mode (Sterling, 2012). One related emphasis is on the evolutionary primacy of reafferent processes in which sensing the world became intertwined with responsivity to the organism’s own actions (Jékely et al., 2021). A further stipulation of embodiment is that meaning is not prespecified in information that is “picked up” by the organism, but rather that embodied action transforms the objective world into the world that the individual experiences (Overton, 2008). Meaning is therefore shaped by the embodiment of the organism, including the kind of body that it has and the nature and development of its capacities for action (Marshall, 2016).

## Embodiment and the Meaning in Life

The evolution of complex animal bodies had particular implications for embodiment, with the capacity for more sophisticated action bringing a new way of being in the world. Evolving nervous systems facilitated the coordination and intertwining of moving, sensing, and acting as a single unit, establishing a new kind of “body-self” (Jékely et al., 2021). The distinction between self and non-self became sharper, bringing to the organism a new kind of perspective or point of view. However, although the evolution of the animal body represented a new chapter in the book of embodiment, it did not begin an entirely new volume. By virtue of the particular organizational properties of living systems, the thread of embodiment runs through all living things. This fundamental point features prominently in a line of theorizing around embodiment known as *autopoietic enactivism* that has its origins in the work of Maturana and Varela (1987; Varela, 1979). In turn, this line of thinking has connections to systems-organizational frameworks in biology (Rosen, 1991) as well as to philosophical lines of inquiry that focus on the particular self-determining properties of living things (Merleau-Ponty, 1967).

The autopoietic enactivist view begins from the premise that living things actively self-maintain themselves through the constant regeneration of the conditions that are necessary to sustain their material existence. This organizational feature of life was termed *autopoiesis* by Maturana and Varela, who focused on the individual cell as a unit enclosed by a semipermeable membrane that acts as a boundary between the inside of the cell and the surrounding medium. The inside of the cell is characterized by chemical reactions and transformations that both generate the components of which the cell is composed and maintain the organization of the cell (its boundary and contents) in the face of entropic tendencies to dissolve that organization. What then defines living beings as unities is their autopoietic organization, such that “it is in this autopoietic organization that they become real and specify themselves at the same time” (Maturana and Varela, 1987, p. 48). This self-specification confers on living organisms a particular kind of autonomy that has been termed *constitutive autonomy* by Froese et al. (2007), in contrast to *behavioral autonomy*, where the identity of the system is imposed externally by an operator or observer. There are clear connections here to the teleology of Kant, who proposed that living things exist for themselves in a way that is different from nonliving entities. As framed by Witherington (2014), from the Kantian perspective the living organism “serves as its own cause, organizing and producing itself such that it causes and results from itself. In this way, living systems constitute natural ends or purposes” (p. 27; see also Farnsworth, 2018).

Returning to the framework of Overton (2008), these considerations take us from the standpoints of bodily morphology and the engaged body to the standpoint of the body as lived experience. In short, constitutive autonomy confers the individual organism with a perspective that arises naturally from the organization of living things and the way that this organization comes about. This notion of the organism having an individual perspective then raises questions about phenomenology–questions that have historically been pushed aside by the computational machine metaphor of the organism that originated in the split ontology of Descartes. The machine metaphor does not allow the question of “What is it like to be a living thing?” to even arise. In contrast, embodiment allows us to consider living beings as having what Maturana and Varela called a “biological phenomenology” that arises through their organizational properties. In this view, biological phenomenology does not go against physical phenomenology: There is no split. Maturana and Varela simply propose that as different classes of unities, living beings and nonliving things such as rocks or snowflakes specify particular phenomenologies. The organization that characterizes nonliving unities is not autopoietic, thus precluding them from having a perspective in the sense of the biological phenomenology of living systems that arises through their inherent autopoietic self-specification. This line of thinking remains an important background framework for studying the emergence of agency and autonomy in living systems, including research that probes the interface of mesoscale physical and physiochemical processes that are common to nonliving and living things and agentive behaviors of cellular systems that are unique to living organisms (Arias Del Angel et al., 2020).

## Implications for Developmental Science

In this brief perspective piece, we have outlined a wide-angle view of embodiment that draws together work across developmental and evolutionary biology, developmental psychology, and embodied cognitive science. Considering the evolution of bodies highlights how embodiment is not an add-on to the study of development, but rather that the origins of development are the origins of embodiment. In turn, bodily morphology cannot be divorced from the active agency of the individual, such that embodied engagement of the developing organism comes to the fore. Within developmental science, this emphasis recalls Piagetian notions of schemes that are shaped through assimilation and accommodation, with the process of equilibration moving the developing organism from one world of meaning to another (Di Paolo, 2019). A renewed focus on these ideas, combined with an emphasis on the self-determination of living systems, can return the study of organization and systems to the center of developmental science (Marshall, 2013, 2014). In particular, the interplay between organization (or structure) and process must be deeply considered (Witherington and Heying, 2015). We note that these themes are emphasized in the metatheoretical approach of process-relational developmental systems theory (Overton and Lerner, 2014; Overton, 2015). This approach places embodiment as a core developmental construct, and is founded in a relational worldview that rejects the separation of life and mind that originated with Descartes yet continues to influence contemporary cognitive science.

A further implication of living things as self-determined concerns the perspective of the developing organism. With respect to human development, the view of embodiment outlined here speaks to the importance of an intraindividual, lifespan developmental approach, which still remains to be widely considered in contemporary theorizing. It also leads to a variety of questions about the embodied origins of the self (and self-other relations) and about the development of agency in relation to the construction of embodied meaning (Marshall, 2016). Adopting this multidisciplinary, broad view of embodiment presents distinct challenges, but also provides a valuable opportunity for developmental scientists to transcend the boundaries of their individual fields of study to create a new vision for the scientific study of development.

## Author Contributions

This article arose through extensive discussions among PM, TH, and SW. All authors contributed to the writing of the manuscript.

## Conflict of Interest

The authors declare that the research was conducted in the absence of any commercial or financial relationships that could be construed as a potential conflict of interest.

## Publisher’s Note

All claims expressed in this article are solely those of the authors and do not necessarily represent those of their affiliated organizations, or those of the publisher, the editors and the reviewers. Any product that may be evaluated in this article, or claim that may be made by its manufacturer, is not guaranteed or endorsed by the publisher.
